# Efficacy of an Abbreviated Induction Regimen of Amphotericin B Deoxycholate for Cryptococcal Meningoencephalitis: 3 Days of Therapy Is Equivalent to 14 Days

**DOI:** 10.1128/mBio.00725-13

**Published:** 2014-01-28

**Authors:** Joanne Livermore, Susan J. Howard, Andrew D. Sharp, Joanne Goodwin, Lea Gregson, Timothy Felton, Julie A. Schwartz, Catherine Walker, Bill Moser, Werner Müller, Thomas S. Harrison, John R. Perfect, William W. Hope

**Affiliations:** ^a^ Antimicrobial Pharmacodynamics and Therapeutics, Department of Molecular and Clinical Pharmacology, University of Liverpool, Liverpool, United Kingdom; ^b^ The University of Manchester, Manchester Academic Health Science Centre, NIHR Translational Research Facility in Respiratory Medicine, University Hospital of South Manchester NHS Foundation Trust, Manchester, United Kingdom; ^c^ Charles River Laboratories, Davis, California, USA; ^d^ Faculty of Life Sciences, The University of Manchester, Manchester, United Kingdom; ^e^ Research Centre for Infection and Immunity, St. George’s University of London, London, United Kingdom; ^f^ Department of Medicine, Duke University, Durham, North Carolina, USA

## Abstract

Cryptococcal meningoencephalitis is an urgent global health problem. Induction regimens using 14 days of amphotericin B deoxycholate (dAmB) are considered the standard of care but may not be suitable for resource-poor settings. We investigated the efficacy of conventional and abbreviated regimens of dAmB for cryptococcal meningoencephalitis in both murine and rabbit models of cryptococcal meningoencephalitis. We examined the extent to which immunological effectors contribute to the antifungal effect. We bridged the results to humans as a first critical step to define regimens suitable for further study in clinical trials. There were significant differences in the murine plasma-versus-cerebrum dAmB concentration-time profiles. dAmB was detectable in the cerebrum throughout the experimental period, even following the administration of only three doses of 3 mg/kg. dAmB induced a fungistatic effect in the cerebrum with a 2- to 3-log_10_ CFU/g reduction compared with controls. The effect of 3 days of therapy was the same as that of daily therapy for 14 days. There was no evidence of increased numbers of CD3^+^ CD4^+^ or CD3^+^ CD8^+^ cells in treated mice to account for the persistent antifungal effect of an abbreviated regimen. The administration of dAmB at 1 mg/kg/day for 3 days was the same as daily therapy in rabbits. The bridging studies suggested that a human regimen of 0.7 mg/kg/day for 3 days resulted in nearly maximal antifungal activity in both the cerebrum and cerebrospinal fluid. An abbreviated regimen (3 days of therapy) of dAmB appears to be just as effective as conventional induction therapy for cryptococcal meningoencephalitis.

## IMPORTANCE

Cryptococcal meningitis is a significant and neglected infection that is associated with excessive morbidity and mortality. In well-resourced health care settings, induction therapy with at least 2 weeks of amphotericin B deoxycholate (dAmB) is advocated. Multiple clinical studies suggest that dAmB is the drug of choice for cryptococcal meningitis. In many parts of the world where the burden of cryptococcal meningitis is highest, it is infeasible to administer dAmB for prolonged periods. This paper provides the experimental basis for the efficacy of abbreviated regimens of dAmB for cryptococcal meningitis. The concept was explored in two well-validated laboratory animal models of cryptococcal meningitis, and the results were bridged to humans by using a range of mathematical modeling techniques. A 3-day regimen is as effective as the standard 14-day course. An abbreviated regimen is significantly more feasible and may enable better antifungal therapy to be administered to many patients with this frequently lethal disease.

## INTRODUCTION

Cryptococcal meningitis is an important, neglected infection that continues to pose many therapeutic challenges. There are few active drugs, a striking paucity of new agents in developmental pipelines, and problems with the global supply of first-line agents that are considered the standard of care in developed health care settings.

In well-resourced health care settings, effective antifungal regimens and combinations for cryptococcal meningitis are relatively well delineated. Induction regimens consist of 2 weeks of amphotericin B deoxycholate (dAmB; Fungizone) at 0.7 to 1.0 mg/kg/day given intravenously (i.v.) in combination with flucytosine at 100 mg/kg/day given orally or i.v. in four divided doses. A lipid preparation of dAmB may be used, although there is less certainty about the optimal regimen—this has recently been addressed by us, as well as other investigators (1, 2). In resource-poor settings, it is often not possible to administer long courses of i.v. dAmB safely because prolonged parenteral therapy is too expensive and/or there are inadequate resources to monitor for and manage drug-related toxicity. These obstacles are especially pertinent for many parts of sub-Saharan Africa, which has a disproportionate share of the world’s burden of cryptococcal meningitis. Thus, many patients do not receive the best antifungal regimens to ensure that their clinical outcomes are optimized. Therefore, alternative and feasible approaches are urgently required.

One potential approach to achieving better clinical outcomes for cryptococcal meningitis in resource-poor settings is the use of high-dose fluconazole alone or in combination with flucytosine (3). Such a regimen is effective and has the advantage of being orally bioavailable. Unfortunately, the outcomes of induction with fluconazole-based regimens are still inferior to those obtained with dAmB. However, phase II clinical data suggest that the outcome of cryptococcal meningitis following a shortened induction regimen with dAmB in combination with other antifungal agents may be favorable (4). In this study, we examined the efficacy of abbreviated versus prolonged induction regimens of dAmB monotherapy for cryptococcal meningoencephalitis. We used two laboratory animal models of cryptococcal meningoencephalitis to define the pharmacokinetic-pharmacodynamic (PK-PD) relationships in both the cerebrum and cerebrospinal fluid (CSF). We have bridged our results to humans to provide abbreviated regimens that may be feasible to use in even the most resource-poor settings and can be further studied in clinical trials.

## RESULTS

### I.v. and intracisternal inoculation of mice and rabbits, respectively, with *C. neoformans* results in reproducible meningoencephalitis.

**FIG 1:**
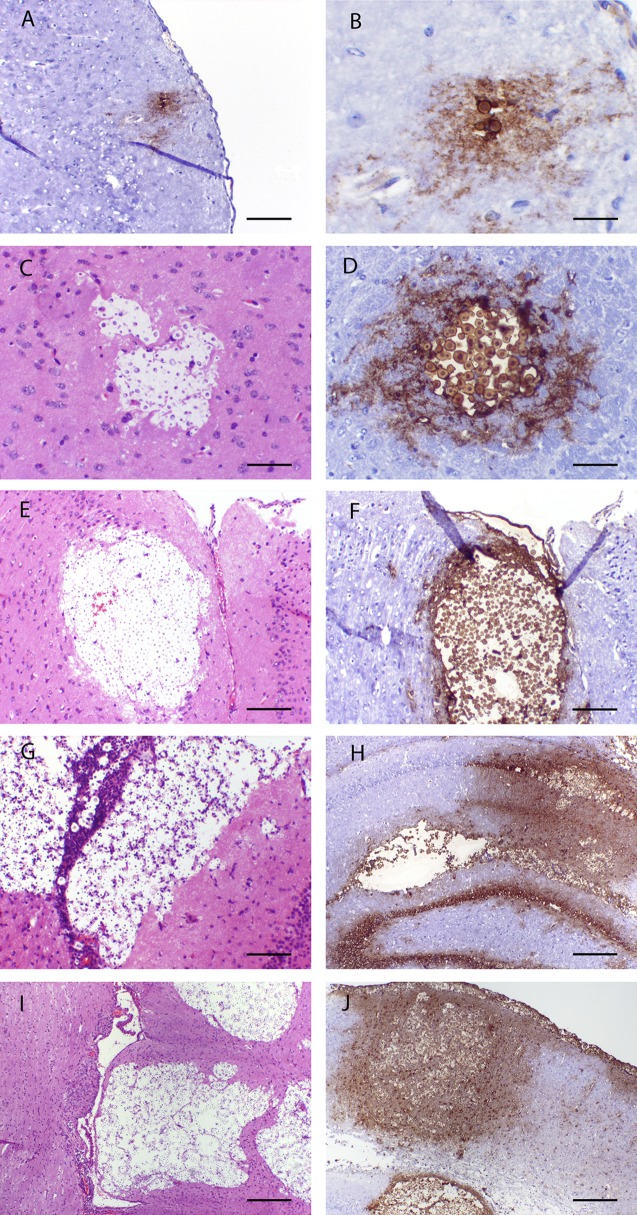
The temporal course of cryptococcal meningoencephalitis in mice inoculated i.v. with *C. neoformans* H99. Small focal lesions containing a small number of organisms early in the infection progressively enlarge throughout the experimental period. There is leaching of capsular material into the surrounding parenchyma. Sections have been stained with hematoxylin and eosin or an anticryptococcal antibody. (A) One hour postinoculation; ×100 magnification; scale bar, 100 µm. (B) One hour postinoculation; ×400 magnification; scale bar, 25 µm. (C) Twenty-four hours postinoculation; ×200 magnification; scale bar, 50 µm. (D) Seventy-two hours postinfection; ×200 magnification; scale bar, 50 µm. (E and F) One hundred twenty hours postinfection; ×100 magnification; scale bars, 100 µm. (G and H) One hundred sixty-eight hours postinfection; ×40 magnification; scale bars, 100 µm. (I and J) Two hundred forty hours postinfection; ×40 magnification; scale bars, 250 µm.

**FIG 2:**
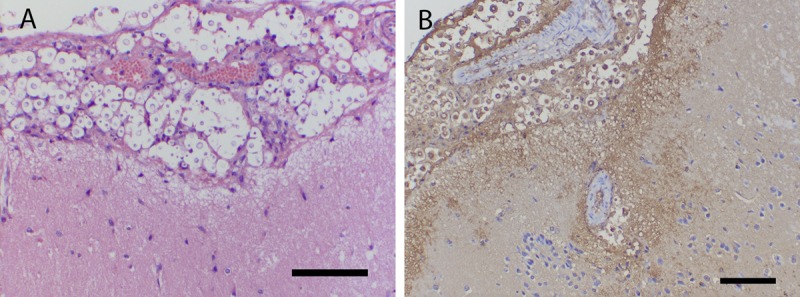
Pathological changes in the meninges of rabbits 7 days postinoculation of 1 × 10^8^ CFU of *C. neoformans* into the cervical cistern. (A) Hematoxylin-and-eosin staining; scale bar, 100 µm. (B) Immunohistochemistry with staining with an anticryptococcal antibody; scale bar, 100 µm.

**FIG 3:**
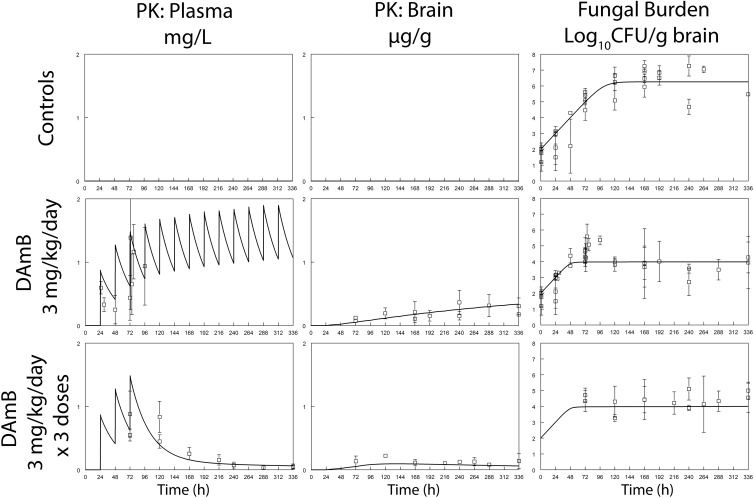
The pharmacokinetics and pharmacodynamics of dAmB at 3 mg/kg against *C. neoformans* in a murine model of cryptococcal meningoencephalitis. There is hysteresis in the plasma-versus-cerebrum pharmacokinetics (PK), with concentrations in the latter progressively increasing throughout the experimental period. The administration of an abbreviated regimen results in persistently detectable concentrations in both plasma and the cerebrum, which accounts for the persistent antifungal effect. For reference, the MIC for the isolate of *C. neoformans* used in these experiments was 0.125 mg/liter. Data are the mean ± standard deviation of four mice. The solid line is the fit of the mathematical model to the data.

**FIG 4:**
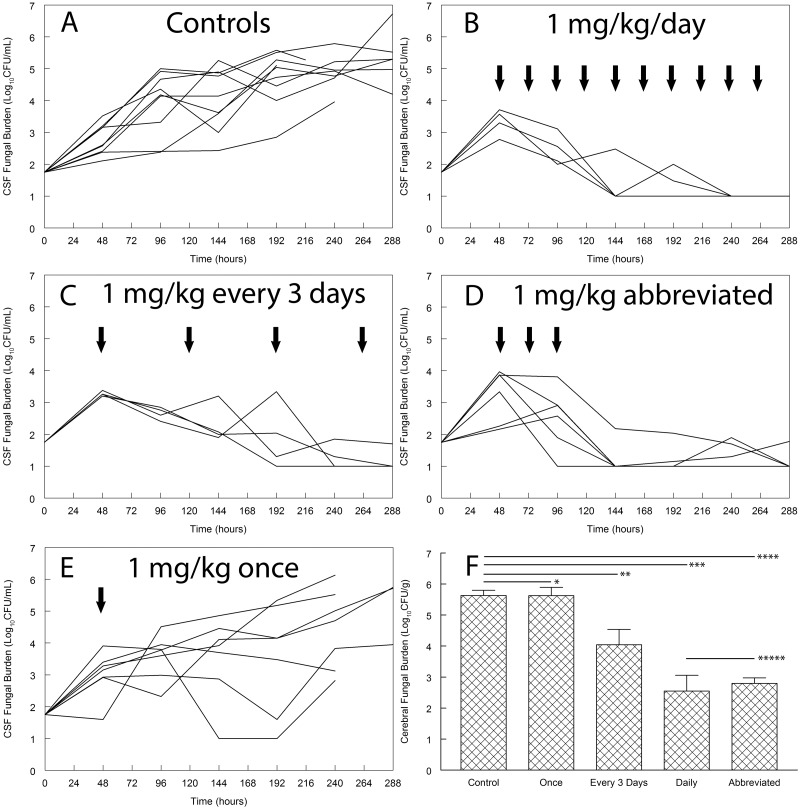
Effects of various regimens of dAmB for experimental cryptococcal meningoencephalitis in rabbits. dAmB at 1 mg/kg was administered daily (B), every third day (C), as an abbreviated regimen for 3 consecutive days (D), or once (E). The solid arrows show the times of drug administration. The data in panels A to E represent the time course of infection in the CSF. Each line represents the fungal burden in an individual rabbit. Panel F shows the fungal density in the cerebrum at the end of the experiment. Each bar represents the mean fungal density ± the standard error of the mean. There is no statistically significant difference in cerebral fungal density between rabbits that received daily therapy and those that received an abbreviated regimen of 1 mg/kg at 48, 72, and 96 h after inoculation. *, *P* = 1.00 (not significant); **, *P* = 0.006; ***, *P* < 0.001; ****, *P* < 0.001; *****, *P* = 1.00 (not significant).

There were progressive histopathological changes throughout the cerebra of mice (Fig. 1). In the initial 24 h postinoculation, there were relatively few yeast cells visible in their cerebra, even though there was strong staining with an anticryptococcal antibody (Fig. 1). In rabbits, the meninges were densely infiltrated by organisms (Fig. 2). There was a logarithmic increase in the fungal burden in the cerebra of untreated mice throughout the first week, which then plateaued at approximately 6 to 7 log_10_ CFU/g cerebrum (Fig. 3). The estimate from the mathematical model for *POPMAX* (the maximum fungal density in the cerebrum) was 6.26 log_10_ CFU/g (Tables 1 and 2). Similarly, there was a progressive logarithmic increase in the fungal density in the CSF of rabbits, which increased in most of the animals to 5 to 6 log_10_ CFU/ml at the end of the experiment (Fig. 4). The rate of increase was slower than that observed in mice (the *K*_*g*_*max* values for rabbits and mice were 0.063 and 0.096 log_10_ CFU/g/h, respectively [Tables 1 and 2]). Interestingly, the intracisternal inoculation of rabbits with *Cryptococcus neoformans* also resulted in reproducible encephalitis with a mean ± standard error of the mean fungal burden of 5.63 ± 0.17 log_10_ CFU/g cerebrum (Fig. 4F).

**TABLE 1:** Mean parameter values and standard deviations for mice and rabbits^*a*^

Parameter	Mean (SD)	
	Mice	Rabbits
*Ka* (h^−1^)	3.07 (0.15)	
*SCL* (liters/h)	0.0018 (0.00007)	0.32 (0.16)
*Vc* (liters)	0.01 (0.00028)	1.64 (1.00)
*K*_*12*_ (h^−1^)	2.53 (0.046)	1.12 (1.24)
*K*_*21*_ (h^−1^)	29.29 (0.16)	29.45 (0.60)
K_23_ (h^−1^)	1.10 (0.09)	1.88 (1.27)
*K*_*32*_ (h^−1^)	0.004 (0.87 × 10^−10^)	0.042 (0.015)
*K*_*cp*_ (h^−1^)	21.90 (0.59)	21.56 (4.27)
*K*_*pc*_ (h^−1^)	2.87 (0.31)	10.72 (9.06)
*Vbrain* (liters)	0.60 (0.69)	0.077 (0.028)
*K*_*g*_*max* (log_10_ CFU/g/h)	0.096 (0.008)	0.063 (0.028)
*Hg*	4.68 (1.52)	5.99 (5.43)
*C*_50_*g* (mg/liter)	0.02 (0.006)	0.154 (0.073)
*POPMAX* (CFU/g cerebrum)	1,822,010 (655,459)	2,776,560 (4,271,150)
*K_k_max* (log_10_ CFU/g/h)		0.043 (0.043)
*Hk*		9.48 (5.66)
*C*_50_*k* (mg/liter)		0.178 (0.035)
Initial condition (CFU/g cerebrum)	101.20 (2.28)	57.15 (38.54)

^a^ See the text for definitions of parameter values.

**TABLE 2:** Mean and median parameter values and estimates of the standard deviations for the population pharmacokinetic model fitted to data obtained from healthy volunteers receiving dAmB at 0.6 mg/kg^*a*^

Parameter	Mean	Median	SD
*SCL* (liters/h)	1.34	1.33	0.09
*V*_*c*_ (liters)	30.8	26.89	5.25
*K*_*cp*_ (h^−1^)	0.11	0.15	0.015
*K*_*pc*_ (h^−1^)	0.05	0.15	0.0024

^a^ See the text for definitions of parameter values.

### Pharmacokinetics and pharmacodynamics of dAmB.

The pharmacokinetics of dAmB in mice and rabbits were well accounted for by linear models. The most striking feature of the pharmacokinetics of dAmB in mice was the extent of hysteresis between plasma and the cerebrum (Fig. 3). The concentration of dAmB progressively accumulated over the experimental period. The prolonged mean residence time within the cerebrum also accounted for the persistence of dAmB in the cerebrum following the administration of only three doses (Fig. 3). A serial-sacrifice experimental design was not possible with rabbits, meaning that it was not possible to obtain robust estimates of the extent of drug penetration of various subcompartments of the central nervous system or the time course of concentrations at those sites. Nevertheless, it was possible to detect dAmB in both the cerebra and meninges of at least some rabbits at the end of the experiment (data not shown).

dAmB induced a dose-dependent decline in the fungal burden within the cerebra of mice. A dosage of 0.1 mg/kg/day had little effect compared with that in untreated controls, but the administration of 1 and 3 mg/kg/day resulted in a 2- to 3-log decline in fungal density relative to that in controls. The pharmacodynamics of dAmB at 3 mg/kg/day in mice are shown in Fig. 3. There was no clear evidence of “fungicidal” antifungal activity within the cerebrum (i.e., there was no evidence of a progressive decline in fungal density) throughout the experimental period. Rather, dAmB resulted in the prevention of inexorable fungal growth within the cerebrum. The antifungal effects of daily dosing and an abbreviated regimen of 3 days were comparable (Fig. 3). The data and mathematical model suggested that this observation could be explained by concentrations of dAmB that exceeded a threshold in the cerebrum. The estimated *C*_50_*g* (i.e., the concentration of dAmB in the cerebrum at which the suppression of growth is half-maximal) was 0.02 µg/g. For both daily administration and the abbreviated regimen, this concentration was readily achieved throughout the experimental period with a dosage of 3 mg/kg/day.

Only a single dosage of 1 mg/kg/day was studied in rabbits—this was based on numerous previous studies of dAmB in rabbits. In those studies, after a single dose of dAmB, CSF levels of 2 to 10 ng/ml were measured over 24 h. Following multiple daily dosing (6 days), the concentrations were approximately 6 ng/ml (5). A daily regimen of dAmB at 1 mg/kg resulted in prompt fungicidal activity within the CSF (Fig. 4B). Comparable fungicidal activity was observed with alternative regimens of 1 mg/kg administered every 3 days and an abbreviated regimen of 1 mg/kg administered 48, 72, and 96 h postinoculation. There was some antifungal activity in at least some rabbits that received a single dose of dAmB, but in all cases there was progressive fungal growth in the CSF in the latter parts of the experimental period (Fig. 4E). There were clear differences in the pharmacodynamics in the CSF and the cerebrum. Despite a rapid decline and apparent sterilization of the CSF in many rabbits, there was persistent fungal infection in the cerebrum. The abbreviated regimen resulted in a fungal burden in the cerebrum comparable to that obtained with daily dosing (*P* = 1.00; Fig. 4F). All treatment arms had a statistically significantly lower fungal burden in the cerebrum than the controls.

### The persistent antifungal activity of an abbreviated dAmB regimen is not caused by immunological effectors.

**FIG 5:**
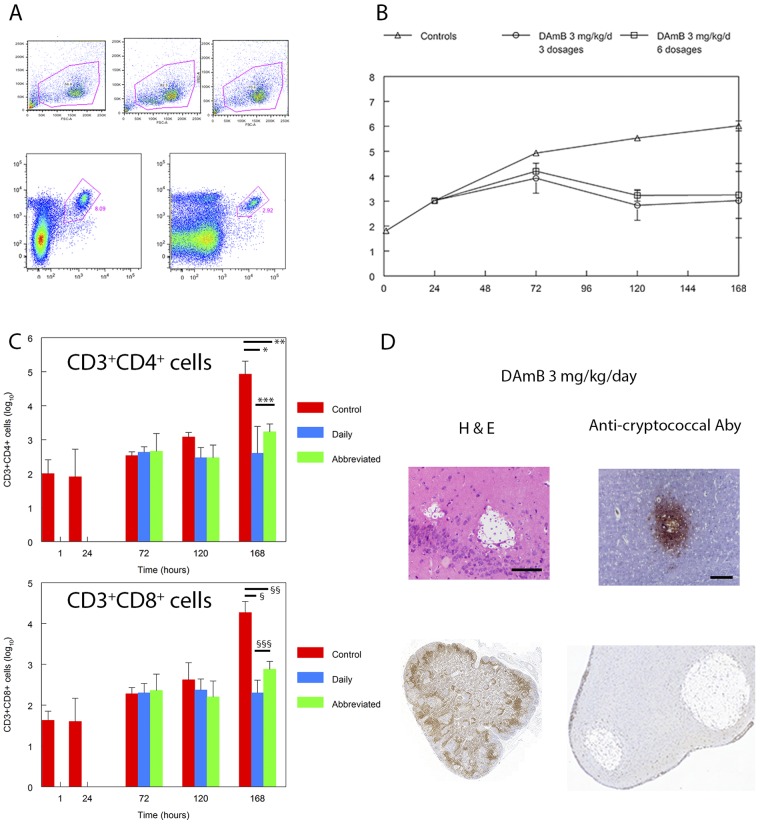
The effect of an abbreviated regimen of dAmB is not due to an immune-mediated effect. Panel A shows the gating strategy; panel B shows the time course of fungal density in the cerebrums of mice infected with *C. neoformans*. Progressive logarithmic growth was seen in controls (open triangles). The antifungal effect of an abbreviated regimen of dAmB at 3 mg/kg (open circles) is comparable to daily dosing with 3 mg/kg (open squares). Data are the mean ± the standard deviation of three or four mice. (C) The absolute numbers of CD3^+^ CD4^+^ and CD3^+^ CD8^+^cells in the cerebrums of mice infected with *C. neoformans*. There was a progressive increase in both CD3^+^ CD4^+^ and CD3^+^ CD4^+^cells in untreated infected controls but no increase in drug-treated mice. *, *P* < 0.001; **, *P* = 0.003; ***, *P* = 0.190 (not significant); §, *P* < 0.001; §§, *P* = 0.002; §§§, *P* = 0.103 (not significant). (D) No evidence of an inflammatory infiltrate in mice treated with dAmB (top two micrographs; scale bars, 25 and 50 µm, respectively) or in infected untreated controls using immunohistochemistry (bottom right micrograph). The bottom left micrograph is a positive control.

The percentages of CD3^+^ CD4^+^ and CD3^+^ CD8^+^ cells increased in the cerebrum in the first 7 days of infection (Fig. 5). Treatment of mice with dAmB at 3 mg/kg/day as both a daily and abbreviated course resulted in a lower absolute number of T cells in the cerebrum than in controls. There was some evidence of an inflammatory infiltrate in untreated controls 7 days postinoculation (for example, Fig. 1G). Studies using flow cytometry suggested that there was a progressive increase in both CD3^+^ CD4^+^ and CD3^+^ CD8^+^ cells in the first week of infection (Fig. 5). At the end of the experiment, there were significantly fewer T cells in the cerebra of treated mice than in those of untreated controls (Fig. 5). There were no differences in the number of CD3^+^ CD4^+^ or CD3^+^ CD8^+^ cells following treatment with the daily versus the abbreviated regimen (*P* = 0.190 and 0.103 for CD3^+^ CD4^+^ and CD3^+^ CD8^+^ cells, respectively; Fig. 5C). Following treatment with dAmB, there was no evidence of an inflammatory infiltrate on hematoxylin-and-eosin staining or following immunohistochemical staining for CD3^+^ cells (Fig. 5D).

### Abbreviated induction regimens of dAmB are predicted to be effective in humans.

**FIG 6:**
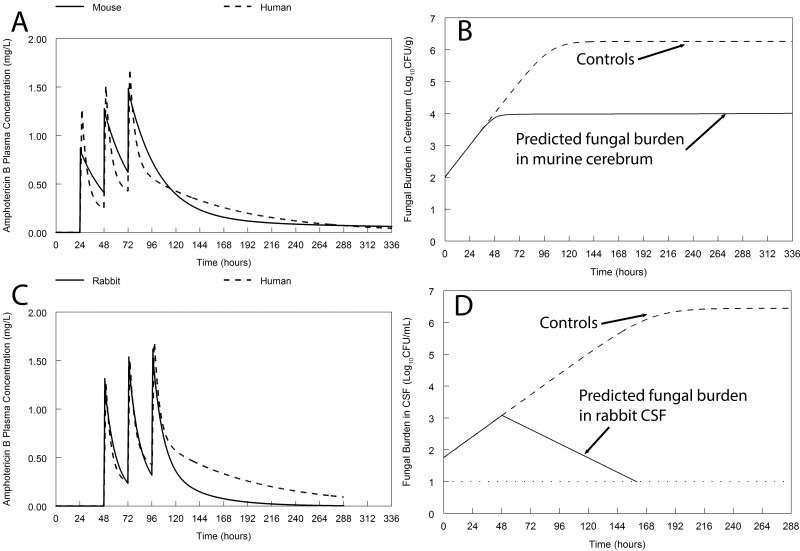
Bridging from experimental models (mice and rabbits) to humans. The murine pharmacokinetics (A) and rabbit pharmacokinetics (C) have been “humanized” so they approximate a human receiving dAmB at 0.7 mg/kg. The predicted pharmacodynamic output from the respective mathematical models for mice (B) and rabbits (D) is shown. The pharmacodynamic outputs for mice and rabbits are the fungal densities in the cerebrum and CSF, respectively. The effect relative to controls is shown. The broken line at the bottom of panel D is the limit of detection (1 log_10_ CFU/ml).

An abbreviated human regimen of dAmB at 0.7 mg/kg administered for 3 days was predicted to result in the suppression of fungal growth within the human cerebrum for at least 2 weeks (Fig. 6). This is a result of the long terminal half-life of dAmB (65.19 h) and the implied hysteresis within a deep and slowly equilibrating tissue site within the central nervous system. Similarly, this same human regimen was predicted to result in prompt fungicidal activity in the CSF (Fig. 6).

## DISCUSSION

Cryptococcal meningoencephalitis is a persistent global health problem (7). There have been a number of studies conducted in resource-poor settings that have attempted to identify optimal induction regimens for cryptococcal meningitis (for examples, see references 3, 8, 9, and 10). Collectively, these studies suggest that dAmB is the most potent fungicidal agent of the currently available compounds. An initial period of 2 weeks of induction therapy is generally used, although different durations have not been rigorously investigated. Longer-term therapy with all dAmB formulations is potentially limited by adverse effects such as nephrotoxicity (decreased renal function and tubular toxicity) and anemia (11). These adverse events may preclude the use of otherwise optimal regimens of dAmB and thereby compromise clinical outcomes. Shorter induction regimens with dAmB may be just as effective and less toxic and enable its use in resource-poor settings. The present study suggests that there is a threshold concentration that is required for antifungal activity within the central nervous system.

dAmB has been extensively studied for cryptococcal meningitis. The first report of successful therapy was in 1957 (6). Very early studies used a dosage of 1 to 1.5 mg/kg/day. While the duration of therapy was often not specified, a total cumulative dose of 3 g was often advocated. Subsequently, there has been a progressive understanding and refinement of regimens of dAmB that are safe and effective. Initially, a lower dAmB dosage of 0.3 mg/kg was studied in combination with flucytosine in an attempt to maximize efficacy and minimize dose-related toxicity (12). More recently, dosages of 0.7 to 1 mg/kg/day have been studied (8, 9). A higher dosage is more effective but also more toxic. Despite all of these studies that have focused on safe and effective regimens, the most appropriate duration of dAmB administration for induction therapy has not been rigorously defined.

In resource-poor health care settings, an abbreviated regimen of dAmB may be feasible and potentially improve the clinical outcome of patients with cryptococcal meningitis. An abbreviated regimen of 5 to 7 days in combination with other antifungal agents has been studied and appears effective (13). Such an approach may have a number of advantages in terms of reduced cost and reduced toxicity and promote its use in resource-poor health care settings. dAmB could potentially be given for 3 days without intensive monitoring of renal function and electrolytes. Patients could be immediately switched to fluconazole, or alternatively, an abbreviated regimen of dAmB could be administered on a “fluconazole backbone.”

The reason an abbreviated regimen of dAmB appears to be effective can be understood on pharmacokinetic grounds. On the basis of the experimental evidence obtained with mice, it is unlikely that an immune-mediated antifungal effect is responsible for its persistent antifungal activity following cessation of dosing. The murine pharmacokinetic studies suggest that dAmB progressively accumulates in the brain despite plasma concentrations being at a “steady state” (i.e., there was evidence of significant hysteresis). The physicochemical properties of dAmB may enable it to “stick” in the lipid-rich cerebrum and not be readily released back into the circulation. Drug concentrations could conceivably be higher in focally abnormal areas in the parenchyma, where there is undoubtedly disruption of the blood-brain-barrier (for example, Fig. 1). As expected, we could not reliably measure amphotericin in the CSF of rabbits. The drug was detectable within the meninges of at least some rabbits but not in a reliable manner. This is potentially because of the friable nature of this tissue and concentrations that are relatively close to the analytical sensitivity of the assay. Nevertheless, the meninges are the subcompartment that is likely to be the most relevant for a detailed understanding of the efficacy of dAmB for cryptococcal meningitis in both rabbits and humans.

The conclusions of this study are predicated on comparable dAmB penetration of the various subcompartments of the central nervous system (i.e., the cerebrum and meninges) in laboratory animal models and humans. Definitive clinical data that enable such an estimate are unlikely to be forthcoming because of obvious ethical constraints (a brain biopsy is not a routine component of clinical care for patients with cryptococcal meningitis). Autopsy studies may be possible but are difficult to conduct, and the quality of the data obtained is often dubious. Such studies enable only a point estimate of penetration (i.e., a simple ratio of plasma-to-brain drug concentrations). These inherent limitations are some of the reasons we chose to use two experimental models of cryptococcal meningoencephalitis to address the pharmacodynamics of an abbreviated regimen of dAmB.

This study highlights pharmacodynamic differences between the cerebrum and CSF. Cryptococcal meningitis is more accurately referred to as meningoencephalitis to reflect the fact that there is involvement of the meninges and CSF, as well as the cerebral parenchyma (14). The rabbit studies suggest that these subcompartments are distinct in terms of their response to dAmB. Both daily and abbreviated regimens of dAmB result in prompt fungicidal activity in the CSF, but there is a significant residual burden within the cerebral parenchyma. The clinical importance of this finding is not clear. While it is possible that a portion of morbidity and mortality is related to suboptimally treated parenchymal disease, results of clinical trials suggest that the rate of decline of yeast in the CSF is an important prognostic marker and is associated with overall survival (9, 15).

Clinical studies are now required to further investigate the utility of shortened dAmB-based induction regimens in the most resource-limited centers, where fluconazole currently remains the mainstay of therapy, building on prior phase II trials (4, 13) and one ongoing phase III trial comparing 7 days with the standard 14 days of dAmB-based induction (ISRCTN45035509). These shortened regimens could be administered as monotherapy or in combination with other antifungal agents. These studies could incorporate the clinical response and death as endpoints to further investigate the effect of the duration of dAmB therapy on the outcome. This study is a further example of the use of PK-PD analyses and bridging studies to generate clinically relevant hypotheses. Such a paradigm is a way that therapeutic advances can be made while minimizing risk.

## MATERIALS AND METHODS

### Organism and MIC.

The challenge strain for all experiments was *C. neoformans* var. *grubii* H99 (ATCC 208821). The organism was stored at −80°C on beads and retrieved prior to each experiment. For rabbit experiments (see below), there were some concerns based on preliminary experiments that the organism had become less virulent as a result of storage. Consequently, the yeast was passaged once in a single mouse. Following i.v. inoculation, the mouse was kept for 10 days before being sacrificed. The brain was removed, homogenized, and plated. The yeast was recovered on Sabouraud agar before being refrozen on beads at −80°C.

For experiments with mice and rabbits, a suspension containing the desired density (1 × 10^8^ CFU/ml) of organisms was prepared in phosphate-buffered saline (PBS; Invitrogen) from yeast cells incubated in 20 ml yeast extract-peptone-dextrose (YPD) broth on an orbital shaker and incubated at 30°C for 48 h.

The MIC of dAmB was determined from three independently conducted experiments using Clinical Laboratory Standards Institute (CLSI) methodology and was 0.125 mg/liter on each occasion.

### Laboratory animal models of cryptococcal meningitis.

All laboratory animal models were performed under project licenses PPL40/3101 (The University of Manchester, Manchester, United Kingdom) and 40/3630 (University of Liverpool, Liverpool, United Kingdom). The protocols used were approved by ethics committees at the respective institutions.

A previously described murine model of cryptococcal meningoencephalitis was initially used to define the pharmacokinetic and pharmacodynamic relationships of dAmB (16). Mice were inoculated i.v. with 3.8 × 10^5^ organisms via the lateral tail vein in 0.25 ml PBS (Invitrogen, Paisley, United Kingdom). The desired inoculum was checked with quantitative cultures. No immunosuppression was used at any point in the experiment since mice are innately susceptible to the development of disseminated cryptococcosis.

Key experimental observations in mice were confirmed in a previously described rabbit model of cryptococcal meningoencephalitis (17), with minor modification of the agents used to induce general anesthesia (see below). Briefly, male New Zealand White rabbits weighing approximately 2 kg were used. Rabbits were inoculated with a cryptococcal suspension directly into the cisterna magna. A target of 1 × 10^8^ CFU in a 1-ml volume was administered. Rabbits were immunosuppressed with cortisone acetate at 7.5 mg/kg given intramuscularly (i.m.), which was started on day −1 relative to inoculation and then administered daily throughout the experimental period. CSF (approximately 1 ml) was removed at 48, 96, 144, 192, 240, and 288 h postinoculation via an intracisternal tap with a 23-gauge needle. Plasma samples for pharmacokinetic analyses were obtained from the lateral ear vein. All procedures were performed under general anesthesia, which was induced with fentanyl-fluanisone (Hypnorm) at 0.3 ml/kg given i.m., followed by diazepam at 0.375 mg/kg. Naloxone at 0.1 mg/kg was used as a reversal agent if required.

### Drug.

dAmB was diluted to the desired concentration with 5% glucose. The specified dosage was administered to mice intraperitoneally (i.p.). Therapy was initiated at 24 h postinoculation and administered every 24 h thereafter for the designed duration of therapy. The dosage effects of dAmB at 0.1, 1, and 3 mg/kg were studied. dAmB was administered to each rabbit i.v. as a slow push via the lateral ear vein. Treatment was commenced 48 h after the intracisternal inoculation of yeast cells and administered every 24 h. On the basis of multiple previous experimental studies of dAmB in rabbits, a single clinically relevant regimen of 1 mg/kg/day was studied (18). The duration of therapy varied from experiment to experiment.

### Pharmacokinetic and pharmacodynamic studies with mice and rabbits.

A serial-sacrifice design was used to estimate the murine pharmacokinetics and pharmacodynamics of dAmB. Three or four mice were used for each dose and time point. Plasma was obtained by terminal cardiac puncture, which was performed under anesthesia with 5% isoflurane (Baxter, Newbury, United Kingdom). Samples were collected throughout the first and fourth dosing intervals at 1, 3, 6, and 24 h and as trough concentrations at later time points. After sacrifice with pentobarbitone, the brain was removed and sectioned in a sagittal plane. One cerebral hemisphere was used to quantify the fungal burden, while the other was used to estimate cerebral concentrations of dAmB. Each half of the sectioned brain was placed in two separate sterile plastic bags. For the pharmacodynamic experiments, the brain was homogenized by using direct pressure and then serially diluted in PBS. These 10-fold dilutions were then plated onto Sabouraud agar and incubated at 30°C for 48 to 72 h to enable the fungal burden to be estimated. The other half was frozen at −80°C to enable the quantification of drug concentrations.

The fungal burden in the CSF of rabbits was estimated by placing serial samples of CSF on YPD agar (preliminary experiments suggested that the recovery and rate of growth of yeast cells were better on YPD than on Sabouraud agar). Plasma samples to enable the estimation of plasma pharmacokinetics were obtained on days 1 and 7. At the end of the experiment, all rabbits were sacrificed and their brains were removed. Their cerebra were homogenized and submitted for quantitative cultures.

### Measurement of dAmB concentrations in plasma and the cerebrum.

DAmB concentrations in plasma (mouse and rabbit) and cerebrum (mouse) samples were measured by high-performance liquid chromatography with a Shimadzu Prominence (Shimadzu) and a Varian Pursuit C_18_ 5-µm column (50 by 2.0 mm; Varian Inc.). The injection volume was 75 µl. A standard curve was constructed in the respective matrix from stock solutions of dAmB (Sigma) at 1,000 mg/liter in methanol (Fisher Scientific). The internal standard was piroxicam at 0.1 mg/liter (Sigma). The starting mobile phase was 80% A, consisting of 1% (vol/vol) aqueous formic acid (Fisher Scientific), and 20% B, consisting of 1% (vol/vol) formic acid in acetonitrile (Fisher Scientific). A gradient was used with progression over 9.5 min from the starting conditions to 30% solution A. The flow rate was 0.8 ml/min. DAmB and the internal standard were detected by using UV light at 385 nm; they eluted after 4.1 and 2.3 min, respectively. The coefficient of variation was <6% over a concentration range 0.1 to 100 mg/liter. The limit of detection was 0.05 mg/liter. The intra- and interday variations were <8%.

### Histology and immunohistochemistry.

The cerebrum was fixed in 10% neutral buffered formalin, cryoprotected in sucrose, and embedded in OCT freezing medium, and 5-µm-thick sections were cut. Tissue sections were treated with peroxidase solution (Envison+ kit; DakoCytomation, Carpinteria, CA) to block endogenous peroxidase activity. A 1.5% goat serum protein block was applied to reduce nonspecific binding. Sections were incubated with an anti-*Cryptococcus* monoclonal antibody (1:1,000 dilution; Mybiosource, San Diego, CA). Slides were reacted with a peroxidase-labeled anti-mouse polymer (Envison+ kit; DakoCytomation, Carpinteria, CA). Antibody-antigen complexes were visualized by using a 3,3′-diaminobenzidine chromogen substrate (DakoCytomation, Carpinteria, CA) for the peroxidase reaction. Slides were counterstained with hematoxylin (Richard-Allan Scientific, Kalamazoo, MI).

Immunohistochemical analysis to visualize CD3^+^ cells in the cerebrum was performed by a previously described method. Briefly, murine cerebrum was fixed in xylene and dried in ethanol before being blocked with peroxidase (Dako Real Peroxidase Blocking Solution). Sections were incubated with a rabbit anti-human CD3^+^ polyclonal antibody (Dako A0452), which is a pan-T-cell marker for the detection of normal and neoplastic T cells that cross-reacts with murine T cells (19). Slides were then reacted with a peroxidase-labeled anti-rabbit polymer (Envison+ System-HRP kit; Dako). Antibody-antigen complexes were visualized by using a 3,3′-diaminobenzidine chromogen substrate for the peroxidase reaction.

### Immunological response to infection.

The immunological response to disseminated cryptococcal infection in the cerebrum was investigated to estimate its potential contribution to the antifungal effect. These experiments were performed only with mice—the necessary antibodies for flow cytometry and immunohistochemistry are readily available for mice but not for rabbits.

Mice were sacrificed by exposure to a rising concentration of CO_2_. Subsequently, blood was replaced after death with PBS that was calcium and magnesium free. The brain was excised and cut into small pieces with a scalpel. The cerebrum was digested with 3 ml of a 0.2-mg/ml collagenase solution (Sigma) and DNase (200 U/brain; Sigma) at 37°C for 30 min. The reaction was stopped by the addition of 1:100 EDTA (0.5 M), followed by incubated for a further 5 min at 37°C. The cell suspension was washed and cells were suspended in 37% Percoll and underlayered with 70% Percoll. The sample was centrifuged at 600 × *g* for 30 min. Lymphocytes isolated from the interface were washed and resuspended in RPMI medium.

Cells were blocked with Fc block (BD Biosciences) for 15 min at 4°C. Cells were washed in FACS wash (PBS, 1% fetal calf serum, 0.1% sodium azide) and then incubated with an antibody mixture (CD3e-fluorescein isothiocyanate [145-2C11; eBioscience, Hatfield, United Kingdom], CD4-APC [RM4-5; eBioscience], and CD8-PE-Cy5 [53-6.7; BD Biosciences]) for 30 min at 4°C. Cells were washed and resuspended in FACS fix (PBS, 1% paraformaldehyde) before being analyzed with an LSRII flow cytometer (BD Biosciences) and FlowJo software (TreeStar Inc., Ashland, OR).

### Mathematical modeling.

The mathematical model was fitted to the entire murine data set by using a population methodology. The nonparametric adaptive grid (NPAG) program (20) was used. The pharmacokinetic and pharmacodynamic data were weighted after initially fitting the same model by using the maximum-likelihood estimator in ADAPT 5 (21) (as previously described—see, for example, reference 22). The structural mathematical model consisted of the following six inhomogeneous differential equations:

(1) $$\frac{dX(1)}{dt}=B\left(1\right)-Ka\times X(1)$$

(2) $$\frac{dX(2)}{dt}=-\left(\frac{SCL}{Vc}+K_{cp}+K_{ce}\right)\times X\left(2\right)+Ka\times X(1)+K_{ec}\times X\left(3\right)+K_{pc}\times X(5)$$

(3) $$\frac{dX(3)}{dt}=-K_{ec}\times X(3)+K_{ce}\times X\left(2\right)-K_{eb}\times X\left(3\right)+K_{be}\times X(4)$$

(4) $$\frac{dX(4)}{dt}=K_{eb}\times X\left(3\right)-K_{be}\times X(4)$$

(5) $$\frac{dX(5)}{dt}=-K_{pc}\times X\left(5\right)+K_{cp}\times X(2)$$

(6) $$\frac{dN}{dt}=K_{g}max\times \left[1-\left(\frac{{\frac{X(4)}{\text{Vbrain}}}^{Hg}}{{\frac{X(4)}{\text{Vbrain}}}^{Hg}+C_{50}g^{Hg}}\right)\right]\times \left(1-\frac{N}{POPMAX}\right)\times N$$

Where *X*(1), *X*(2), *X*(3), *X*(4), and *X*(5) represent the amounts of dAmB (milligrams) in the peritoneum, central compartment, cerebral endothelium, central tissue, and peripheral compartment, respectively. *B*(1) represents the injection of dAmB (milligrams) into the peritoneums of the mice; *Ka* is the first-order rate constant connecting the peritoneal cavity with the central compartment; *K*_*ce*_, *K*_*ec*_, *K*_*eb*_, *K*_*be*_, *K*_*cp*_, and *K*_*pc*_ represent the first-order rate constants connecting the various compartments; *K*_*g*_*max* is the maximum rate of growth in the brain; *Vbrain* is the volume of the brain; *C*_50_*g* is the concentration of dAmB in the brain at which there is half-maximal inhibition of growth; *POPMAX* is the maximum theoretical density of organisms in the brain; *N* is the number of organisms in the cerebrum; and *Hg* is the slope function. *SCL* is the clearance of drug (liters/h), and *Vc* is the volume of central compartment (liters).

Equation 1 describes the movement of drug out of the peritoneum and into the central compartment (i.e., plasma). Equation 2 describes the movement of dAmB into and out of the central compartment (plasma). Equations 3 and 4 describe the movement of the drug into deep compartments within the brain. Equation 5 describes the movement of the drug into and out of the peripheral compartment (i.e., everything except the brain). Equation 6 describes the pharmacodynamics of dAmB. This equation contains terms that describe capacity-limited fungal growth in the brain and the drug-induced suppression of fungal growth.

Several points about the construction and fitting of this mathematical model to the murine data deserve emphasis. (i) The plasma and brain pharmacokinetic data were discordant (i.e., there was hysteresis), which required several deep pharmacokinetic compartments to enable this to be described; and (ii) we did not observe fungicidal activity in the cerebrum. Therefore, we did not include a term that described a decline in log_10_ CFU/g (as opposed to rabbits [see below]).

A similar mathematical model was used to describe the PK-PD data from rabbits, although there were some differences from mice. Most notably, (i) dAmB was administered as an i.v. push directly into the central compartment. (ii) There were no temporal measurements of dAmB in the cerebra or meninges of rabbits (and dAmB was not detectable in the CSF); consequently, we did not have good estimates of the shape of the concentration-time profile of dAmB within the central nervous system. (iii) The equation describing the pharmacodynamics of dAmB included an extra term that described drug-induced fungal killing (i.e., fungicidal activity). The equation describing the drug’s pharmacokinetics and pharmacodynamics in rabbits is as follows:

$$\frac{dN}{dt}=K_{g}max\times \left[1-\left(\frac{{\frac{X\left(2\right)}{Vbrain}}^{Hg}}{{\frac{X(2)}{Vbrain}}^{Hg}+C_{50}g^{Hg}}\right)\right]\times \left(1-\frac{N}{POPMAX}\right)\times N-K_{kill}max\times \left(\frac{{\frac{X(2)}{Vbrain}}^{Hk}}{{\frac{X(2)}{Vbrain}}^{Hk}+C_{50}k^{Hk}}\right)\times N$$

Where *X*(2) is the amount of dAmB in the central compartment (milligrams). There is an extra term that describes the dAmB-induced killing. *K*_*kill*_*max* is the maximum rate of drug-induced killing, *Hk* is the slope function, *Vbrain* is the volume of the brain, and *C*_50_*k* is the concentration of the drug in the brain at which killing is half-maximal.

### Bridging from mice and rabbits to humans.

The pharmacokinetics of dAmB in adult volunteers were determined from previously published data (23). Five patients received 0.6 mg/kg of dAmB as an i.v. infusion once-over a 2-h period. A standard two-compartment pharmacokinetic model was fitted to the data by using NPAG. The fit of the model to the data was assessed by using the same criteria as described above for the laboratory animal models of infection.

The clinical implications of the experimental data were explored by humanizing the murine and rabbit pharmacokinetics *in silico*. The program BestDose was used to estimate the dosages needed to be administered to both mice and rabbits that would result in a plasma pharmacokinetic profile equivalent to that for humans receiving 0.7 mg/kg. The mathematical models fitted to mice and rabbits were then used to estimate the antifungal effect of each regimen.

### Statistics.

Differences in the fungal burden in the cerebra of rabbits and the number of T cells in the cerebra of mice were determined by analysis of variance with a Bonferroni correction as appropriate. All calculations were performed with SYSTAT version 11.
